# Pathways linked to unresolved inflammation and airway remodelling characterize the transcriptome in two independent severe asthma cohorts

**DOI:** 10.1111/resp.14302

**Published:** 2022-06-07

**Authors:** Stephany Sánchez‐Ovando, Stelios Pavlidis, Nazanin Zounemat Kermani, Katherine Joanne Baines, Daniel Barker, Peter G. Gibson, Lisa G. Wood, Ian M. Adcock, Kian Fan Chung, Jodie Louise Simpson, Peter A.B. Wark

**Affiliations:** ^1^ Priority Research Centre for Healthy Lungs, Faculty of Health and Medicine University of Newcastle Newcastle New South Wales Australia; ^2^ Data Science Institute Imperial College London London UK; ^3^ Faculty of Health and Medicine University of Newcastle Newcastle New South Wales Australia; ^4^ Respiratory and Sleep Medicine John Hunter Hospital NSW New Lambton Heights New South Wales Australia; ^5^ National Heart and Lung Institute Imperial College London London UK

**Keywords:** airway remodelling, biopsies, gene expression, inflammation, pathogenesis, severe asthma, sputum, transcriptome

## Abstract

**Background and objective:**

Severe asthma (SA) is a heterogeneous disease. Transcriptomic analysis contributes to the understanding of pathogenesis necessary for developing new therapies. We sought to identify and validate mechanistic pathways of SA across two independent cohorts.

**Methods:**

Transcriptomic profiles from U‐BIOPRED and Australian NOVocastrian Asthma cohorts were examined and grouped into SA, mild/moderate asthma (MMA) and healthy controls (HCs). Differentially expressed genes (DEGs), canonical pathways and gene sets were identified as central to SA mechanisms if they were significant across both cohorts in either endobronchial biopsies or induced sputum.

**Results:**

Thirty‐six DEGs and four pathways were shared across cohorts linking to tissue remodelling/repair in biopsies of SA patients, including SUMOylation, NRF2 pathway and oxidative stress pathways. MMA presented a similar profile to HCs. Induced sputum demonstrated *IL18R1* as a shared DEG in SA compared with healthy subjects. We identified enrichment of gene sets related to corticosteroid treatment; immune‐related mechanisms; activation of CD4^+^ T cells, mast cells and IL18R1; and airway remodelling in SA.

**Conclusion:**

Our results identified differentially expressed pathways that highlight the role of CD4^+^ T cells, mast cells and pathways linked to ongoing airway remodelling, such as IL18R1, SUMOylation and NRF2 pathways, as likely active mechanisms in the pathogenesis of SA.

## Abstract 2

Transcriptome analysis from endobronchial biopsies and induced sputum from two independent cohorts of adults with severe asthma (SA) (U‐BIOPRED and Australian NOVocastrian Asthma cohort) demonstrated shared differentially expressed pathways previously linked to persistent unresolved inflammation and novel mechanisms of airway remodelling, which may represent potential novel mechanistic pathways involved in the pathogenesis of SA.

**See related** editorial

## INTRODUCTION

Severe asthma (SA) is a complex disease with diverse clinical and inflammatory presentations.^1^ Asthma guidelines recommend phenotyping SA patients to identify responders to targeted therapy.^1^ Both inflammation and airway remodelling are contributing factors to the heterogeneous pathogenesis and severity.^2^ Most studies based on clinical or transcriptomic profiles have largely demonstrated inflammatory mechanisms and few have examined the links to airway remodelling in SA. Previous studies have identified Type‐2 T‐helper cell (Th2)‐based mechanisms in epithelial brushings^3^ and sputum,^4^ as well as non‐Th2 pathways associated with sputum^5^ and biological pathways have been identified, associated with persistent airflow limitation^6^ and age of onset.^7^ However, mechanisms driving structural airway changes remain largely unknown, and they are not addressed by current treatments.^8^ A better understanding of the mechanisms driving inflammatory and remodelling features is necessary to define more personalized and precise management of SA and may even contribute to the discovery of new therapies. Despite our increasing awareness of a wide range of mechanisms involved in SA, there have been no comprehensive analyses to identify mechanisms shared by more than one independent cohort of patients with SA.

The aim of this study was to identify differentially expressed genes (DEGs), altered pathways and gene sets, shared by two independent cohorts of non‐smoking adults with SA, compared with mild/moderate asthma (MMA) and healthy controls (HCs). We hypothesized that the independent cohorts will share DEGs and pathways compared with MMA and HC. In this study, we used clinical and transcriptomic data obtained from endobronchial biopsies and induced sputum from the European Unbiased Biomarkers for the Prediction of Respiratory Outcomes (U‐BIOPRED) study^9^ and the Australian Priority Research Centre for Healthy Lungs – NOVocastrian Asthma cohort (NOVA).^5^, ^10^

## METHODS

### Study design

This is a comparison of two cross‐sectional cohorts of adults with asthma. Details of the design and study populations of U‐BIOPRED^9^, ^11^, ^12^ and NOVA have been described elsewhere.^5^, ^13^

### SA definition

Participants from both cohorts had confirmed diagnoses of asthma and met the definition of SA (see Appendix S1 in the Supporting Information). Participants with SA and MMA were non‐smokers for at least the past 12 months with less than 5 packs/year. Participants with MMA had controlled or partially controlled asthma symptoms, whilst receiving a dose of <500 μg fluticasone propionate/day or equivalent. HCs had no history of asthma or wheeze, had no other chronic respiratory disease and were non‐smokers for at least the past 12 months with a smoking history of ≤5 packs/year and pre‐bronchodilator forced expiratory volume in 1 s was ≥80% predicted.

### Statistical analysis

Data were analysed using Stata 15 (StataCorp, TX) and reported as means and SD for normally distributed data or median and interquartile range (Q1–Q3). Comparisons were made using either a Wilcoxon signed‐rank or a Student's *t*‐test, depending on the outcome distribution. Fishers' exact test was used for categorical data.

U‐BIOPRED transcriptomic profiling was performed using the GeneChip® Human Genome U133 Plus 2.0 microarray (Affymetrix, Santa Clara, CA) and the NOVA cohort used Illumina's HT‐12 version 4 Beadchips (details have been published previously).^12^, ^14^ Data are available from Gene Expression Omnibus public database (https://www.ncbi.nlm.nih.gov/geo, accession numbers GSE76227, GSE76262, GSE147878 and GSE147880, respectively). For details on clinical and array data analysis, pathway identification and gene set variation analysis (GSVA), see Appendix S1 and Table S1 in the Supporting Information.

## RESULTS

### Cohort characteristics

Characteristics of participants of U‐BIOPRED and NOVA cohorts are presented in Table 1. In both cohorts, those with SA had at least moderate post‐bronchodilator airflow obstruction and were on regular treatment with high‐dose inhaled corticosteroid (ICS, median‐dose 1000 μg/day). Participants with SA from U‐BIOPRED cohort had worse asthma control, and a higher proportion of them were chronic users of oral corticosteroids than in the NOVA cohort. In both cohorts, those with MMA were similar in terms of sex and smoking history. Participants with MMA in U‐BIOPRED were younger than SA participants, but in the NOVA cohort, MMA and SA were of similar age. HCs in both cohorts were younger than MMA and SA participants.

**TABLE 1:** Characteristics of participants in U‐BIOPRED and NOVocastrian Asthma cohorts

	U‐BIOPRED				NOVA			
	SA	MMA	HC	*p*‐value	SA	MMA	HC	*p*‐value
*N*	91 (44.6)	42 (20.5)	38 (18.6)	—	42 (35.9)	36 (30.8)	23 (19.7)	—
Sex, F, *n* (%)	56 (61.5)^a^	24 (57.1)	13 (34.2)	0.02	23 (54.8)	27 (75.0)	14 (60.9)	0.17
Age (years), mean (SD)	51 (13)^b^ ^,^ ^c^	41 (14)	39 (14)	<0.001	58 (13)	57 (15)	44 (16)	0.23
BMI (kg/m^2^), mean (SD)	28.8 (5.6)^b^	26.4 (4.7)	25.4 (3.3)	<0.001	32.0 (7.0)	31.5 (8.6)	25.5 (4.7)	0.41
Smoking history, *n* (%)				0.57				0.82
Never smoker	73 (80.2)	36 (85.7)	33 (86.8)		37 (88.1)	29 (80.6)	20 (87.0)	
Ex‐smoker	18 (19.8)	6 (14.3)	5 (13.2)		1 (2.4)	7 (19.4)	3 (13.0)	
Current smoker	0 (0)	0 (0)	0 (0)		0 (0)	0 (0)	0 (0)	
Pack‐years, median (IQR)	1.5 (0.9, 2.6)	3.8 (0.3, 4.3)	0.7 (0.2, 1)	0.29	4 (4, 4.8)	1.7 (0.4, 3.5)	5 (2.9, 5)	0.20
ICS dose mg, median (IQR)	—	—	—	—	1000 (1000, 1000)^d^	0 (0, 250)	N/A	<0.001
OCS use, *n* (%)	34 (37.4)	0	0	—	4 (9.5)	0	0	—
OCS dose mg, median (IQR)	10 (7.5, 15)	0	N/A	0.09	7.5 (5, 12.5)	0	N/A	0.71
ACQ, mean (SD)	4.5 (1.6)	3.7 (2.6)	N/A	0.04	2 (1.2)^d^	0.9 (0.7)	N/A	<0.001
FEV_1_ % predicted, mean (SD)	65.7 (22.8)^b^ ^,^ ^c^	91.2 (15.2)^b^	101.1 (12.8)	<0.001	74.9 (20.5)^b^	82.4 (18.9)^b^	102.5 (10.4)	<0.001
FEV_1_/FVC, mean (SD)	60.9 (14.2)^b^ ^,^ ^c^	73.3 (7.6)^b^	78.5 (6.4)	<0.001	66.2 (10.8)^b^	72.6 (9.0)	80.4 (4.6)	0.01
Endobronchial biopsy, *n*	30	22	22	—	34	18	13	—
Induced sputum, *n*	51	14	12		8	18	10	
Both, *n*	10	6	4		0	0	0	

Abbreviations: ACQ, Asthma Control Questionnaire; FEV_1_, forced expiratory volume in 1 s; FVC, forced vital capacity; HC, healthy control; ICS, inhaled corticosteroid; IQR, interquartile range; MMA, mild/moderate asthma; OCS, oral corticosteroid; SA, severe asthma.

^a^ *p* ≤ 0.05 versus HC.

^b^ *p* ≤ 0.05 versus HC.

^c^ *p* ≤ 0.05 versus MMA.

^d^ *p* ≤ 0.05 versus MMA.

### Differentially expressed genes

**FIGURE 1:**
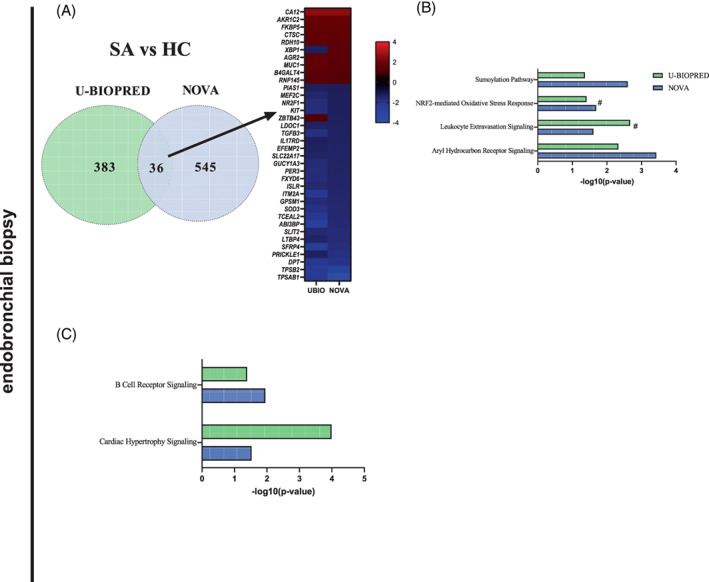
Shared differentially expressed genes and canonical pathways for SA contrasts in endobronchial biopsies. (A) SA versus HC (pathways depicted have false discovery rate ≤0.05). Colours represent different cohorts: U‐BIOPRED (green) and NOVocastrian asthma (NOVA) (blue). (B) SA compared with HC. (C) MMA compared with HC. HC, healthy control; MMA, mild/moderate asthma; NOVA, NOVocastrian Asthma cohort; SA, severe asthma; UBIO, U‐BIOPRED cohort.

Differential expression analysis of the three groups (SA, MMA and HC) was performed using endobronchial biopsies and induced sputum for both cohorts, and shared DEGs (false discovery rate ≤0.05) were identified by overlapping lists of genes (Figure 1).

In endobronchial biopsies, when SA was compared with HC, there were 36 shared DEGs between the U‐BIOPRED and NOVA cohorts (Figure 1A). No shared genes were identified when MMA was compared with SA or HC in both cohorts. No shared genes were identified in induced sputum. List of DEGs can be found in Tables S2 and S3 in the Supporting Information.

### Canonical pathway analysis

In endobronchial biopsies, there were four shared canonical pathways identified in SA compared with HC in the U‐BIOPRED and NOVA cohorts: SUMOylation pathway, NRF2‐mediated oxidative stress response, leucocyte extravasation signalling and aryl hydrocarbon receptor signalling (Figure 1B). There were two shared canonical pathways in MMA compared with HC: B‐cell receptor signalling and cardiac hypertrophy signalling (Figure 1C). Details of the canonical pathways can be found in Tables S4–S7 in the Supporting Information. No shared canonical pathways were identified in induced sputum.

### Gene set variation analysis

**FIGURE 2:**
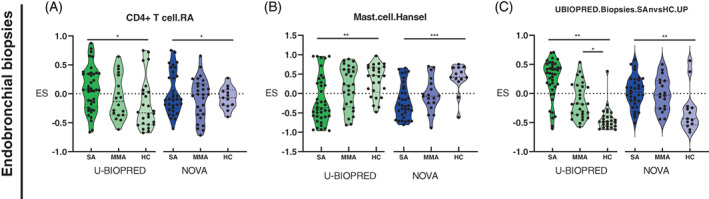
Immunological gene sets identified in endobronchial biopsies of participants with SA smokers/ex‐smokers compared with HCs. (A) CD4^+^ T‐cell set (CD4^+^ T cell.RA); (B) mast cell gene set (Mast.cell.Hansel); and (C) U‐BIOPRED biopsies gene set comprising upregulated genes in SA (UBIOPRED.Biopsies.SAnvsHC.UP). Colours represent different cohorts: U‐BIOPRED (green scale) and NOVocastrian asthma (NOVA) (blue scale). ES: enrichment score ranging from −1 to 1; HC, healthy control; SA, severe asthma. ***p ≤ 0.001, **p ≤ 0.01, *p ≤ 0.05.

**FIGURE 3:**
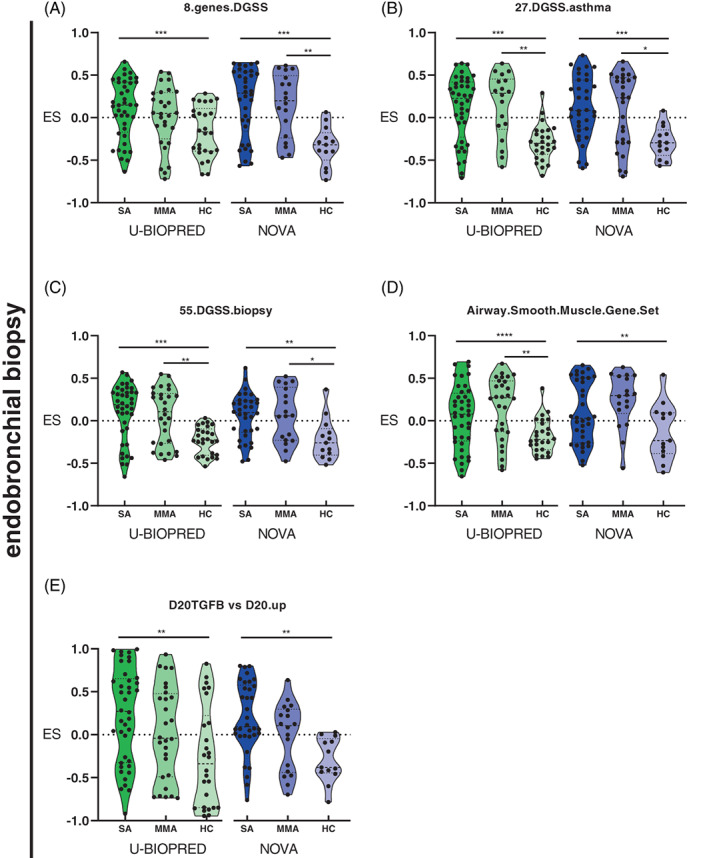
Gene sets associated with steroid response identified in endobronchial biopsies of non‐smoker participants with SA compared with HCs. (A) Set of eight genes from glucocorticoid a gene set; (B) lung development gene set (developmental glucocorticoid gene set [DGGS]) associated with asthma; (C) DGGS associated with steroid response in bronchial biopsies of participants with asthma; (D) airway smooth muscle set following treatment with dexamethasone; and (E) fibroblasts treated with transforming growth factor‐β. Colours represent different cohorts: U‐BIOPRED (green scale) and NOVA (blue scale). ES, enrichment score ranging from −1 to 1; HC, healthy control; SA, severe asthma. ****p ≤ 0.0001, ***p ≤ 0.001, **p ≤ 0.01, *p ≤ 0.05.

In endobronchial biopsies, there were eight gene sets significantly enriched in SA compared with HC in both cohorts; three were associated with immunological mechanisms (Figure 2A), four with steroid response (Figure 3A–D) and one with mechanism of airway remodelling (Figure 3E). Immunological gene sets shared between cohorts were: (i) CD4^+^ T‐cell gene set found enriched in SA compared with HC (Figure 2A); (ii) a mast cell gene set^15^ significantly less enriched in SA compared with HC (Figure 2B); and (iii) a U‐BIOPRED signature reported upregulated in bronchial biopsies in SA. We also identified higher enrichment of these gene sets in SA compared with HC in the NOVA cohort (Figure 2C). No significantly enriched gene sets were identified in SA compared with MMA in either cohort. Shared gene sets associated with steroid treatment and enriched in SA compared with HC include: (i) a glucocorticoid gene set^16^ (Figure 3A); (ii) a developmental glucocorticoid gene set (DGGS)^16^ (Figure 3B); (iii) a DGGS known to be modulated in asthma biopsies, following treatment with ICS (Figure 3C).^16^ In addition, we found significantly less enrichment in SA compared with HC for a (iv) gene set identified in airway smooth muscle following treatment with dexamethasone (Figure 3D).^17^ A shared gene set related to airway remodelling was enriched in SA compared with HC; airway fibroblasts treated with TFG‐β gene set (Figure 3E).^18^ Details of gene sets identified can be found in Table S8 in the Supporting Information.

**FIGURE 4:**
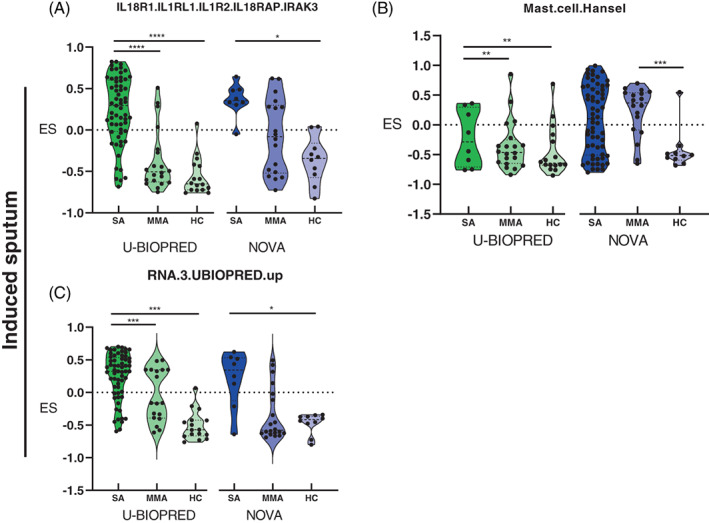
Immunological gene sets identified in induced sputum of participants with SA smokers/ex‐smokers compared with HCs. (A) IL18 receptor 1 set; (B) mast cell gene set; and (C) U‐BIOPRED sputum gene set comprising upregulated genes (RNA.3.UBIOPRED.up). Colours represent different cohorts: U‐BIOPRED (green scale) and NOVA (blue scale). ES, enrichment score ranging from −1 to 1; HC, healthy control; SA, severe asthma. ****p ≤ 0.0001, ***p ≤ 0.001, **p ≤ 0.01, *p ≤ 0.05.

In induced sputum, three gene sets associated with immunological mechanisms were identified enriched across the groups in U‐BIOPRED and NOVA cohorts. Shared gene sets include: (i) IL18R1 gene set enriched in SA compared with HC (Figure 4A); (ii) a mast cell gene set; and (iii) a gene set comprising genes previously reported to be upregulated in SA sputum in the U‐BIOPRED cohort^11^ (Figure 4B,C). Details of gene sets identified can be found in Tables S1 and S9 in the Supporting Information.

## DISCUSSION

This study, for the first time, has compared the transcriptome from endobronchial biopsies and induced sputum of two independent, well‐characterized cohorts of SA to groups with mild to moderate asthma and HCs. We showed that four canonical pathways altered across the two SA U‐BIOPRED and NOVA cohorts: SUMOylation pathway, NRF2‐mediated oxidative stress response, leucocyte extravasation and aryl hydrocarbon receptor signalling. Enriched gene sets in SA were related to response to corticosteroid treatment, immune‐related mechanisms and airway remodelling. In those on high‐dose ICS, we did not demonstrate markers of Type‐2 inflammation; however, we demonstrated dysregulation of SUMOylation, NRF2‐mediated oxidative stress response and IL18R1 pathways strongly linked to tissue damage and repair, which are likely involved in the process of ongoing airway remodelling. Overall, the comparative transcriptomic analysis performed in these two independent cohorts of SA was able to pinpoint novel genes, pathways linked to unresolved inflammation and airway remodelling that may play key roles in the pathogenesis of SA.

We identified overrepresentation of SUMOylation signalling pathway in both SA cohorts. Small ubiquitin‐related modifier (SUMO) family proteins become covalently attached to other proteins, as a post‐translational modification to modify cellular function.^19^ In asthma, SUMOylation has been implicated in airway remodelling,^20^, ^21^ fibroblast transformation and innate immunity, by supressing Type‐1 interferon (IFN) responses,^22^, ^23^, ^24^ and regulation of glucocorticoid receptor function.^25^ PIAS proteins are involved in transcriptional regulation through the SUMO pathway. Among the genes involved in this pathway and identified in SA in the two cohorts studied here is *PIAS1*, an important regulator of nuclear factor kappa B (NF‐κB).^23^ Resulting from cytokine stimulation, the p65 subunit of NF‐κB translocates to the nucleus, binds to PIAS1 and inhibits cytokine‐induced NF‐κB‐dependent gene activation,^23^ promoting transforming growth factor‐β1 (TGF‐β1)‐induced activation of smooth muscle α‐actin.^26^ The NRF2 anti‐oxidant stress pathway is also a target for SUMOylation,^27^ and this interaction has been shown to affect the oxidative stress response to respiratory syncytial virus.^28^ In the mouse, the NRF2 oxidative stress signalling pathway has been shown to contribute to airway inflammation and remodelling, by promoting goblet cell hyperplasia, and hyperresponsiveness in allergen‐mediated asthma.^29^ In our study, we identified overrepresentation of the NRF2‐mediated oxidative stress signalling pathway in SA across both cohorts, suggesting the role of this pathway independent of oxidative stress due to cigarette smoke exposure. These findings show, for the first time, that dysregulation of the SUMOylation and NRF2 pathways may represent a key mediator of mechanisms of airway oxidative stress response and airway remodelling in SA.

In induced sputum, from SA in both cohorts, we identified enrichment of IL18R1, a cytokine receptor for IL‐18, which can induce Th1 and Th2 responses, innate immunity through NK and mast cells,^30^ and play a role in allergic inflammation^31^ and atopic asthma.^32^ Interestingly, IL‐18 induces a cytotoxic response via an IFN‐γ‐dependent mechanism, and fibrotic airway remodelling, mucus metaplasia and vascular remodelling through IL‐17A^33^ and IL‐13.^34^, ^35^, ^36^ In this study, we observed enrichment of the IL18R1 gene set in the sputum of SA, previously described in U‐BIOPRED^37^ and the Severe Asthma Research Program cohorts.^38^ These findings show a potential mechanism linking epithelial dysregulation in driving fibroblast differentiation towards myofibroblasts and mucus production, key processes in airway remodelling^36^ which were active in both SA cohorts.

GSVA identified shared, significantly enriched gene sets across the two cohorts.^6^, ^7^ Gene sets enriched in SA are subsets of a larger set of glucocorticoid genes known to be associated with steroid treatment, lung development, maturation and asthma susceptibility.^16^ There was also enrichment in both SA cohorts for a gene set identified in airway smooth muscle following treatment with dexamethasone. The enrichment of these sets is not surprising given the use of high‐dose ICS in both SA cohorts studied.^17^, ^39^ However, these sets of genes provide potential insights into the mechanisms involved in steroid responsiveness in SA. Shared immunological gene sets include a CD4^+^ T‐cell gene set comprising a subset of genes that are implicated in asthma through IL‐6‐mediated STAT3 signalling, associated with poor lung function^40^ and persistent airflow obstruction in SA.^6^ The shared gene set related to airway remodelling is comprised of genes upregulated in fibroblasts treated with TGF‐β. Among the genes in this gene set is *SPOCK1*, an extracellular proteoglycan that induces epithelial to mesenchymal transition through the TGF‐β1 pathway,^18^, ^41^ with mesenchymal transition being an important mechanism of airway remodelling and *SPOCK1* representing a potential novel target in lung fibrosis.

We found that in the sputum of SA there was high enrichment of a mast cell gene set,^15^ while the same gene set was found less enriched in endobronchial biopsies of SA compared with HC.^15^ Our results support the previous enrichment reported for this gene set in epithelial brushings of SA adult onset,^7^ and highlight the importance of considering compartmentalization of inflammation and mobilization of mast cells in SA. Finally, there were no differentially enriched gene sets identified in SA compared with MMA in either cohort. This result is consistent with previously reported data^6^ and may reflect the possibility that other mechanisms underlie the severity of the disease, mechanisms that may not be identified through gene expression analysis, including lipid and eicosanoid expression or post‐translational modifications.

The strength of our study is that it represents the largest comprehensive examination of transcriptomic profiles in two independent well‐characterized cohorts of SA to date, and that the DEGs, pathways and gene sets presented here provide a novel insight into mechanisms involved in the pathogenesis of SA and the potential development of remodelling that remains active, in spite of treatment with high‐dose corticosteroids and other currently available therapies.

Our study has some limitations. The data are from cross‐sectional cohorts and do not consider the variability of the inflammatory nature of SA. Histological markers of fibrosis and remodelling were unavailable to correlate with airway remodelling genes and pathways identified in this study. In both cohorts, the HC group was younger than SA and MMA groups. Airway narrowing has been previously associated with older age in subjects with fatal asthma; the impact of this difference on airway narrowing was not evaluated in this study. This study utilized endobronchial biopsies to sample diverse components of the airway wall, and therefore the genes and pathways found here reflect this diversity. Future investigation of individual contributions of different cellular components is required. Participants from both cohorts were on high doses of ICS and compliance with treatment was assumed; however, it was not assessed. The number of induced sputum SA samples was limited in the NOVA cohort, and this was reflected in the limited differences observed. In addition, analysis using sputum granulocytes may detect additional mechanistic pathways. The degree of variability in methods of sample collection across cohorts was also not evaluated. Finally, both cohorts were predominantly Caucasian, and the reported results may not reflect important factors in other racial groups with SA.

In conclusion, adults with SA from two independent cohorts shared differentially expressed pathways, SUMOylation, NRF2 pathways, *SPOCK1* and *IL18R1*, which have previously been associated with airway remodelling and fibrosis and that may potentially be involved in the pathogenesis of adult SA. Further analysis of these SA mechanisms is warranted.

## AUTHOR CONTRIBUTION

**Stephany Sánchez‐Ovando:** Conceptualization (lead); data curation (lead); formal analysis (lead); funding acquisition (equal); investigation (lead); methodology (lead); project administration (equal); visualization (lead); writing – original draft (lead); writing – review and editing (lead). **Stelios Pavlidis:** Conceptualization (equal); data curation (equal); formal analysis (equal); methodology (equal); writing – review and editing (equal). **Nazanin Zounemat Kermani:** Methodology (equal); writing – review and editing (equal). **Katherine Joanne Baines:** Conceptualization (equal); formal analysis (equal); funding acquisition (equal); writing – original draft (equal); writing – review and editing (equal). **Daniel Barker:** Methodology (equal); writing – review and editing (equal). **Peter G. Gibson:** Conceptualization (equal); writing – review and editing (equal). **Lisa G. Wood:** Conceptualization (equal); writing – review and editing (equal). **Ian M. Adcock:** Conceptualization (equal); funding acquisition (equal); investigation (equal); writing – original draft (equal); writing – review and editing (equal). **Kian Fan Chung:** Conceptualization (equal); funding acquisition (equal); investigation (equal); writing – original draft (equal); writing – review and editing (equal). **Jodie Louise Simpson:** Conceptualization (equal); formal analysis (equal); funding acquisition (equal); investigation (equal); writing – original draft (equal); writing – review and editing (equal). **Peter A.B. Wark:** Conceptualization (equal); formal analysis (equal); funding acquisition (equal); investigation (equal); writing – original draft (equal); writing – review and editing (equal).

## CONFLICTS OF INTEREST

Ian M. Adcock was supported by the EU‐IMI (FP7/2007–2013), Wellcome Trust (093080/Z/10/Z), EPSRC (EP/T003189/1) and by the UK MRC (MR/T010371/1). Kian Fan Chung is on advisory boards for GlaxoSmithKline, AstraZeneca, Novartis, Boehringer Ingelheim and Teva; is part of GlaxoSmithKline Scientific Board of the Clean Breathing Institute; and has received payment for lectures from AstraZeneca, Novartis, Roche and Merck. Peter G. Gibson is supported by GlaxoSmithKline and has received payment for lectures from AstraZeneca, GlaxoSmithKline, Sanofi and Novartis. The remaining authors declare no competing interests.

## HUMAN ETHICS APPROVAL DECLARATION

The NOVA study was approved by the University of Newcastle (H‐163‐1205) and Hunter New England Human Research Ethics Committee (05/08/10/3.09). The U‐BIOPRED study was approved by the ethics committee of each participating clinical institution, and adhered to the standards set by International Conference on Harmonisation and Good Clinical Practice. Written informed consent was obtained from all participants of both cohorts. Clinical Trial registration: NCT01982162 at  ClinicalTrials.gov.

## Supporting information


**Appendix S1.** Supporting information.

**Table S1.** Gene sets entered into gene set variation analysis.

**Table S2.** Endobronchial biopsies.
**Table S3.** Induced sputum.

**Table S4.** SA versus HC—endobronchial biopsy.
**Table S5.** MMA versus HC—endobronchial biopsy.
**Table S6.** SAN versus HC—induced sputum.
**Table S7.** SA versus MMA—induced sputum.

**Table S8.** U‐BIOPRED and NOVA—endobronchial biopsies: gene set variation difference in mean enrichment score and statistical comparison.
**Table S9.** U‐BIOPRED and NOVA—induced sputum: gene set variation difference in mean enrichment score and statistical comparison.

## Data Availability

Gene expression data are publicly available at Gene Expression Omnibus (https://www.ncbi.nlm.nih.gov/geo; accession numbers: GSE76227 and GSE76262, details in Methods section). Additional data from this study are available from the corresponding author upon request.
