# Supplementary material for: Pathways linked to unresolved inflammation and airway remodelling characterize the transcriptome in two independent severe asthma cohorts
Source: Respirology. 2022 Jun 7;27(9):730–8. doi: 10.1111/resp.14302 (PMC9540453; doi:10.1111/resp.14302)
Supplement: Supplementary file 1 — Appendix S1. Supporting information. [file RESP-27-730-s004.docx]

**APPENDIX S1**

**Severe asthma diagnosis and definition**

U-BIOPRED participants with asthma had either airflow reversibility (increase in forced expiratory volume in 1s (FEV_1_) > 12% predicted or 200 mL following inhalation of 400 μg salbutamol), airway hyperresponsiveness (methacholine provocative concentration causing a 20% fall in FEV_1_ < 8 mg·mL−1, or diurnal peak expiratory flow amplitude > 8% of mean), or a decrease in FEV_1_ of 12% predicted or 200 mL within 4 weeks after tapering maintenance treatment.

NOVA cohort is assembled from subjects with asthma recruited from 2004 to 2015 that had either airflow reversibility (FEV_1_ > 12% predicted or 200 mL following inhalation of 400 μg salbutamol), airway hyperresponsiveness (bronchial provocation with 4.5% saline solution causing a 15% decline in FEV_1_). We collected data on lung function, asthma symptom control, medication use and history of exacerbation.

In order to compare cohorts, we classified NOVA participants using the SA definition and grouping from U-BIOPRED, as previously defined.^1^ SA was defined as asthma with uncontrolled symptoms and frequent exacerbations (≥2 per year) despite high-dose inhaled corticosteroids (⩾1000 μg fluticasone propionate per day or equivalent dose).

**Ethical considerations**

The NOVA study was approved by the University of Newcastle (H-163-1205) and Hunter New England Human Research Ethics Committee (05/08/10/3.09).  The U-BIOPRED study was approved by the ethics committee for each participating clinical institution, and adhered to the standards set by International Conference on Harmonisation and Good Clinical Practice. It is registered on ClinicalTrials.gov (identifier: NCT01982162). Written informed consent was obtained from all participants in both cohorts.

**Clinical data analysis**

Data were analysed using Stata 15 (StataCorp, Tex) and reported as means and standard deviations (SD) for normally-distributed data or median and interquartile range (Q1-Q3). Comparisons were made using either a Wilcoxon signed-rank or a Student’s *t*-test, depending on the outcome distribution. Fishers’ exact test was used for categorical data.

**Microarray data analysis**

U-BIOPRED transcriptomic profiling was performed using the GeneChip® Human Genome U133 Plus 2.0 microarray (Affymetrix, Santa Clara, CA). Details have been published previously (Gene Expression Omnibus <https://www.ncbi.nlm.nih.gov/geo>; Accession Numbers: GSE76227, GSE76262). The NOVA cohort hybridized a total of 750 ng cRNA to the Illumina’s HT-12 version 4 Beadchips and was scanned using the Illumina Bead Station and captured using BeadScan 3.5.11 (Illumina, San Diego, USA). Raw data were processed using GenomeStudio software as previously described^2^ (Accession Numbers: GSE147878, GSE147880). Data were normalised, and quality control analysed using *lumi* package, with differentially-expressed genes (DEGs) identified using linear models, adjusted by sex and age using a *limma* package (an empirical Bayes method) and moderated Students *t*-test adjusted for multiple comparisons using a Benjamini-Hochberg (significant level of false discovery rate; FDR≤0.05) in R Bioconductor. Pathway analysis was performed using Ingenuity Pathway Analysis (Qiagen, Hilden, Germany) by inputting all significant genes (FDR≤0.05) and fold change values for z-score calculation using the right-tailed Fisher exact test. Significant pathways were identified as having p-value ≤0.05, and z-score of more than or equal to 2, or less or equal to -2. Gene-set variation analysis (GSVA) was used to assess enrichment in gene expression microarray data for a collection of 156 gene-sets with a focus on immune cells, GC responses, and airway remodelling.^3, 4^ Shared DEGs, altered pathways and gene-sets were identified when statistical significance, as previously established above, was recorded in both cohorts.

**Gene-set variation analysis**

Gene-set variation analysis (GSVA) was used to assess enrichment to 156 gene-sets in gene expression microarray data. Sample-wise enrichment scores (ES) irrespectively of group labels were calculated for each participant and for each of the gene-sets based on the gene expression data. ES range from a value of -1 to 1. Mean ES, difference of ES (dES) were calculated and group comparison was performed using one-way ANOVA and multiple comparison test was performed using false discovery rate method, Benjamini and Hochberg. In order to minimize the false discovery, only gene-sets with p–value < 0.05 and a dES ≥ 0.2 were considered significant, following the Microarray Consortium for Quality Control recommendations regarding the need for applying group-difference thresholds in order to stringently limit false discovery.^3^

**REFERENCES**

1 Shaw DE, Sousa AR, Fowler SJ, Fleming LJ, Roberts G, Corfield J, Pandis I, Bansal AT, Bel EH, Auffray C, Compton CH, Bisgaard H, Bucchioni E, Caruso M, Chanez P, Dahlen B, Dahlen SE, Dyson K, Frey U, Geiser T, Gerhardsson de Verdier M, Gibeon D, Guo YK, Hashimoto S, Hedlin G, Jeyasingham E, Hekking PP, Higenbottam T, Horvath I, Knox AJ, Krug N, Erpenbeck VJ, Larsson LX, Lazarinis N, Matthews JG, Middelveld R, Montuschi P, Musial J, Myles D, Pahus L, Sandstrom T, Seibold W, Singer F, Strandberg K, Vestbo J, Vissing N, von Garnier C, Adcock IM, Wagers S, Rowe A, Howarth P, Wagener AH, Djukanovic R, Sterk PJ, Chung KF. Clinical and inflammatory characteristics of the European U-BIOPRED adult severe asthma cohort. The European respiratory journal. 2015; **46**: 1308-21.

2 Baines KJ, Simpson JL, Wood LG, Scott RJ, Gibson PG. Transcriptional phenotypes of asthma defined by gene expression profiling of induced sputum samples. J Allergy Clin Immunol. 2011; **127**: 153-60, 60.e1-9.

3 Hekking PP, Loza MJ, Pavlidis S, De Meulder B, Lefaudeux D, Baribaud F, Auffray C, Wagener AH, Brinkman P, Lutter R, Bansal AT, Sousa AR, Bates SA, Pandis I, Fleming LJ, Shaw DE, Fowler SJ, Guo Y, Meiser A, Sun K, Corfield J, Howarth P, Bel EH, Adcock IM, Chung KF, Djukanovic R, Sterk PJ. Transcriptomic gene signatures associated with persistent airflow limitation in patients with severe asthma. The European respiratory journal. 2017; **50**.

4 Hekking PP, Loza MJ, Pavlidis S, de Meulder B, Lefaudeux D, Baribaud F, Auffray C, Wagener AH, Brinkman PI, Lutter RI, Bansal AT, Sousa AR, Bates SA, Pandis Y, Fleming LJ, Shaw DE, Fowler SJ, Guo Y, Meiser A, Sun K, Corfield J, Howarth PH, Bel EH, Adcock IM, Chung KF, Djukanovic R, Sterk PJ. Pathway discovery using transcriptomic profiles in adult-onset severe asthma. J Allergy Clin Immunol. 2018; **141**: 1280-90.
