# Supplementary material for: Pathways linked to unresolved inflammation and airway remodelling characterize the transcriptome in two independent severe asthma cohorts
Source: Respirology. 2022 Jun 7;27(9):730–8. doi: 10.1111/resp.14302 (PMC9540453; doi:10.1111/resp.14302)
Supplement: Supplementary file 5 — Table S8. U‐BIOPRED and NOVA—endobronchial biopsies: gene set variation difference in mean enrichment score and statistical comparison. Table S9. U‐BIOPRED and NOVA—induced sputum: gene set variation difference in mean enrichment score and statistical comparison. [file RESP-27-730-s002.docx]

**Table S8**. U-BIOPRED and NOVA– endobronchial biopsies: Gene set variation difference in mean enrichment score and statistical comparison.

| **U-BIOPRED** | | | | | | | | | |
| --- | --- | --- | --- | --- | --- | --- | --- | --- | --- |
|  | **SA vs MMA** | | | **SA vs HC** | | | **MMA vs HC** | | |
| **Gene Set** | dES | p-value | adj p-value | dES | p-value | adj p-value | dES | p-value | adj p-value |
| **CD4T.cells.RA** | 0.15 | 0.073 | 0.480 | 0.26 | 0.003 | **0.021** | 0.11 | 0.256 | 0.405 |
| **Mast.cell.Hansel** | -0.32 | 0.013 | 0.259 | -0.52 | 0.000 | **0.002** | -0.20 | 0.161 | 0.312 |
| **8.genes.GCGS.Sharma** | 0.09 | 0.250 | 0.560 | 0.26 | 0.000 | **0.010** | 0.17 | 0.070 | 0.210 |
| **27.DGSS.up.epithelial brushings** | 0.03 | 0.780 | 0.900 | 0.41 | 0.000 | **0.001** | 0.38 | 0.000 | **0.010** |
| **55.DGSS.up.biopsy** | 0.04 | 0.560 | 0.850 | 0.33 | 0.000 | **0.001** | 0.29 | 0.000 | **0.010** |
| **LUNG.ASM.DEX.HS.UP** | -0.03 | 0.568 | 0.925 | -0.24 | 0.000 | **0.000** | -0.22 | 0.000 | **0.002** |
| **UBIOPRED_Biopsies_SAnvsHC** | 0.36 | 0.000 | **0.001** | 0.67 | 0.000 | **0.001** | 0.32 | 0.000 | **0.007** |
| **SIG126.D20TGFBvsD20.DOWN** |  | 0.099 | 0.175 |  | 0.003 | **0.004** |  | 0.063 | 0.155 |
| **SIG132.ASMAsthmavsHealthyAtopicControls.UP** |  | 0.009 | **0.018** |  | 0.001 | **0.004** |  | 0.438 | 0.584 |
| **SIG148.VitD.ASM.FatalAsthmaCoexpressionC11** |  | 0.222 | 0.332 |  | 0.001 | **0.014** |  | 0.058 | 0.173 |
| **NOVA** | | | | | | | | | |
|  | **SA vs MMA** | | | **SA vs HC** | | | **MMA vs HC** | | |
| **Gene Set** | dES | p-value | adj p-value | dES | p-value | adj p-value | dES | p-value | adj p-value |
| **CD4T.cells.RA** | 0.12 | 0.238 | 0.966 | 0.27 | 0.002 | **0.027** | 0.12 | 0.261 | 0.875 |
| **Mast.cell.Hansel** | -0.20 | 0.078 | 0.966 | -0.52 | 0.000 | **0.001** | -0.32 | 0.013 | 0.152 |
| **8.genes.GCGS.Sharma** | 0.02 | 0.829 | 0.909 | 0.46 | 0.000 | **0.001** | 0.43 | 0.001 | **0.019** |
| **27.DGSS.up.epithelial brushings** | 0.08 | 0.350 | 0.560 | 0.41 | 0.000 | **0.001** | 0.32 | 0.000 | **0.050** |
| **55.DGSS.up.biopsy** | -0.01 | 0.980 | 0.990 | 0.26 | 0.000 | **0.010** | 0.26 | 0.000 | **0.050** |
| **LUNG.ASM.DEX.HS.UP** | -0.05 | 0.232 | 0.966 | -0.17 | 0.000 | **0.002** | -0.12 | 0.007 | 0.122 |
| **UBIOPRED_Biopsies_SAnvsHC** | 0.092 | 0.254 | 0.453 | 0.31 | 0.000 | **0.004** | 0.22 | 0.020 | 0.154 |
| **SIG126.D20TGFBvsD20.DOWN** |  | 0.172 | 0.239 |  | 0.002 | **0.009** |  | 0.102 | 0.175 |
| **SIG132.ASMAsthmavsHealthyAtopicControls.UP** |  | 0.586 | 0.703 |  | 0.006 | **0.014** |  | 0.004 | **0.011** |

**Table S9.** U-BIOPRED and NOVA – induced sputum: Gene set variation difference in mean enrichment score and statistical comparison.

| **U-BIOPRED** | | | | | | | | | |
| --- | --- | --- | --- | --- | --- | --- | --- | --- | --- |
|  | **SAn vs MMA** |  |  | **SAn vs HC** |  |  | **MMA vs HC** |  |  |
|  | dES | p-value | adj p-value | dES | p-value | adj p-value | dES | p-value | adj p-value |
| **IL18R1.IL1RL1.IL1R2.IL18RAP.IRAK3** | 0.61 | 0.000 | **0.000** | 0.80 | 0.000 | **0.000** | 0.19 | 0.154 | 0.999 |
| **Mast.cells.Hansel** | 0.44 | 0.002 | **0.005** | 0.53 | 0.001 | **0.002** | 0.09 | 0.623 | 0.999 |
| **RNA.3.UBIOPRED.Sputum.up** | 0.60 | 0.000 | **0.000** | 0.76 | 0.000 | **0.000** | 0.16 | 0.187 | 0.999 |
| **NOVA** | | | | | | | | | |
|  | **SAn vs MMA** |  |  | **SAn vs HC** |  |  | **MMA vs HC** |  |  |
| **Genesets** | dES | p-value | adj p-value | dES | p-value | adj p-value | dES | p-value | adj p-value |
| **IL18R1.IL1RL1.IL1R2.IL18RAP.IRAK3** | 0.44 | 0.008 | 0.388 | 0.73 | 0.000 | **0.023** | 0.29 | 0.053 | 0.343 |
| **Mast.cells.Hansel** | -0.45 | 0.007 | 0.388 | 0.20 | 0.268 | 0.525 | 0.65 | 0.000 | **0.030** |
| **RNA.3.UBIOPRED.Sputum.up** | 0.25 | 0.127 | 0.565 | 0.68 | 0.000 | **0.040** | 0.43 | 0.005 | 0.134 |
